# Multiscale Computational and Pharmacophore-Based Screening of ALK Inhibitors with Experimental Validation

**DOI:** 10.3390/ph18081207

**Published:** 2025-08-15

**Authors:** Ya-Kun Zhang, Jian-Bo Tong, Yue Sun, Yan-Rong Zeng

**Affiliations:** 1 College of Chemistry and Chemical Engineering, Shaanxi University of Science and Technology, Xi’an 710021, China; 2 School of Chinese Ethnic Medicine, Guizhou Minzu University, Guiyang 550025, China

**Keywords:** ALK inhibitor, MTT, virtual screening, molecular docking, molecular dynamics simulations

## Abstract

**Background:** Anaplastic lymphoma kinase (ALK) is a key receptor tyrosine kinase involved in regulating signaling pathways critical for cell proliferation, differentiation, and survival. Mutations or rearrangements of the ALK gene lead to aberrant kinase activation, driving tumorigenesis in various cancers. Although ALK inhibitors have shown clinical benefits, drug resistance remains a significant barrier to long-term efficacy. Developing novel ALK inhibitors capable of overcoming resistance is therefore essential. **Methods:** A structure-based pharmacophore model was constructed using the 3D structures of five approved ALK inhibitors. Systematic virtual screening of the Topscience drug-like database was performed incorporating PAINS filtering, ADMET prediction, and molecular docking to identify promising candidates. In vitro antiproliferative assays, molecular docking, molecular dynamics simulations, and MM/GBSA binding free energy calculations were used to evaluate biological activity and elucidate binding mechanisms. **Results:** Two candidates, F1739-0081 and F2571-0016, were identified. F1739-0081 exhibited moderate antiproliferative activity against the A549 cell line, suggesting potential for further optimization. Computational analyses revealed its probable binding modes and interactions with ALK, supporting the observed activity. **Conclusions:** This study successfully identified novel ALK inhibitor candidates with promising biological activity. The integrated computational and experimental approach provides valuable insights for the rational design of optimized ALK inhibitors to address drug resistance in cancer therapy.

## 1. Introduction

Anaplastic lymphoma kinase (ALK), a critical member of the receptor tyrosine kinase (RTK) family [1], possesses an extracellular domain responsible for specific ligand recognition and mediates the transduction of extracellular signals into intracellular responses [2,3]. Upon ligand binding, ALK undergoes conformational changes that facilitate receptor dimerization and subsequently activate its intrinsic tyrosine kinase activity [4]. Activated ALK phosphorylates various intracellular substrates and regulates multiple downstream signaling pathways, including PI3K/AKT, JAK/STAT, and MAPK/ERK [5,6], which play pivotal roles in maintaining normal cellular processes such as proliferation, differentiation, and survival. Abnormal activation of ALK, resulting from gene fusion, point mutation, or gene amplification, often leads to constitutive kinase activity [7,8]. This persistent activation drives continuous downstream signaling, disrupts cell cycle regulation, inhibits apoptosis, and promotes uncontrolled cell proliferation and tumor progression [9,10]. Dysregulated ALK signaling has been extensively implicated in a range of solid tumors, particularly non-small cell lung cancer (NSCLC) [11], anaplastic large cell lymphoma (ALCL) [6], and neuroblastoma (NB) [12]. Therefore, targeting aberrant ALK activation and developing potent, selective small-molecule inhibitors with favorable pharmacokinetic properties has become a major focus in the field of anticancer drug discovery.

With the clinical approval and widespread application of ALK inhibitors such as Crizotinib, Alectinib, and Ceritinib in the treatment of ALK-positive malignancies, these agents have demonstrated substantial efficacy in delaying tumor progression [13]. Nonetheless, prolonged treatment is frequently accompanied by the emergence of resistance-associated mutations, including L1196M and G1202R [14,15], which significantly impair the binding affinity of inhibitors to the ATP-binding pocket, thereby diminishing therapeutic efficacy [16,17]. L1196M, a prototypical gatekeeper mutation, induces conformational alterations within the kinase binding pocket that increase steric hindrance to inhibitor binding. This mutation substantially diminishes the binding affinity of various inhibitors, including first-generation Crizotinib, and compromises the efficacy of certain second-generation inhibitors, thereby constituting a principal mechanism underlying clinical resistance. The G1202R mutation, characterized by the introduction of a bulkier side chain and altered electrostatic properties, markedly perturbs inhibitor-binding interactions within the kinase domain. This structural alteration substantially compromises the binding affinity of numerous second-generation ALK inhibitors, thereby conferring a high degree of drug resistance [18,19]. Consequently, the rational design and screening of inhibitors specifically targeting L1196M, G1202R, and other resistance-associated mutations represent pivotal avenues of research aimed at enhancing therapeutic efficacy in ALK-positive malignancies.

In recent years, with the continuous development of computer-aided drug design (CADD) technologies, virtual screening, as one of its core methodologies, has been widely applied in the discovery and design of novel targeted inhibitors due to its significant advantages in compound to target recognition efficiency, binding affinity prediction, and lead compound optimization [20]. In this study, a ligand-based pharmacophore conformation-matching virtual screening strategy was employed to systematically screen 50,000 compounds from the Topscience-like druggable database, ultimately identifying 80 molecules with excellent pharmacophore matching features and a Phase Screen Score greater than or equal to 2. Subsequently, through PAINS filtering, ADMET property prediction, and molecular docking, two potential candidate compounds, F1739-0081 and F2571-0016, were successfully selected. Further experimental validation and computational analyses systematically elucidated their possible binding modes and mechanisms of action. This study provides an important theoretical basis for the future development of novel ALK inhibitors capable of overcoming resistance-associated mutations.

## 2. Results

### 2.1. Pharmacophore Modeling Results

A structure-based pharmacophore model was constructed using the structural information of five clinically approved ALK small-molecule inhibitors. The resulting pharmacophore hypothesis encompassed four essential chemical features: two hydrogen bond acceptors, one hydrogen bond donor, and one aromatic ring. The corresponding scoring metrics are detailed in Supplementary Materials Table S1.

Supplementary Materials Table S1 presents a comprehensive evaluation of the pharmacophore mapping performance of five clinically approved ALK inhibitors within the constructed model, focusing on key parameters including drug-likeness alert indices (Stars), spatial conformational compatibility (Volume Score), and overall fit metrics (Fitness/Phase Screen Score). Among the evaluated compounds, Ceritinib demonstrated the highest degree of alignment with the pharmacophore model, as reflected by a Fitness score of 2.326 and a Volume Score of 0.559, indicating a high level of structural complementarity. Its Stars value of 3 suggests that its physicochemical properties associated with drug-likeness remain within an acceptable range.

Brigatinib, Crizotinib, and Alectinib exhibited moderate pharmacophore compatibility, with corresponding Fitness scores of 1.832, 1.419, and 1.322, respectively. All three compounds also displayed Stars values of 3, suggesting a favorable balance between structural fit and drug-likeness, and underscoring their potential utility as reference scaffolds in future optimization efforts. In contrast, Lorlatinib yielded the lowest Fitness score (0.892); despite having a Stars value of 3, its limited spatial and chemical congruence with the pharmacophore features may reflect differences in its binding mode or mechanism of action relative to the other inhibitors.

**Figure 1:**
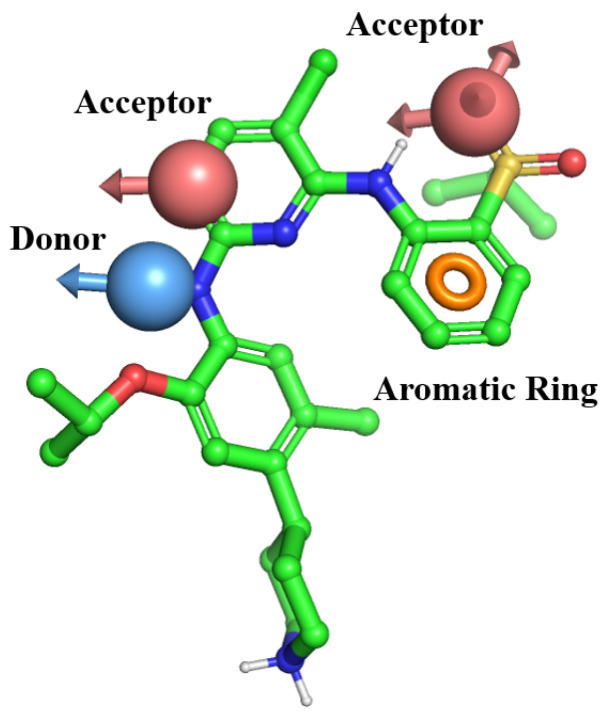
Optimal pharmacophore screening model.

Taken together, compounds with high pharmacophore alignment (Fitness ≥ 1.5) and acceptable drug-likeness profiles (Stars ≤ 3), notably Ceritinib (Figure 1), exhibit a favorable balance between model compatibility and physicochemical properties and thus represent promising structural templates for guiding the identification of candidate compounds in subsequent virtual screening studies.

### 2.2. Optimal Pharmacophore Model Validation Results

**Figure 2:**
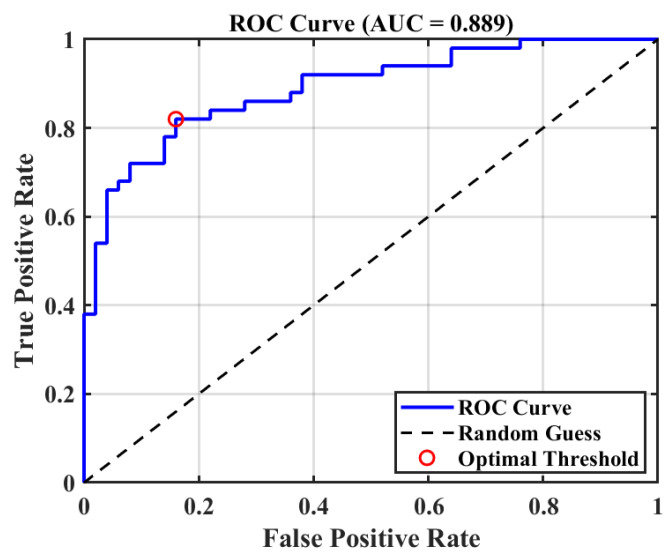
Optimal pharmacophore model validation results.

To rigorously assess the discriminatory capability of the constructed pharmacophore model in differentiating active compounds from inactive counterparts, a receiver operating characteristic (ROC) curve was generated and the corresponding area under the curve (AUC) was computed. As illustrated in Figure 2, the model achieved an AUC of 0.889, markedly surpassing the random classification baseline (AUC = 0.5), thereby demonstrating robust classification performance and satisfactory generalizability. The ROC curve’s proximity to the upper-left corner indicates consistently high sensitivity and specificity across varying discrimination thresholds. Furthermore, the optimal cutoff point yielded a true positive rate of approximately 0.82 and a false positive rate near 0.18, underscoring the model’s high efficacy and accuracy in virtual screening applications. Collectively, these results substantiate the pharmacophore model’s reliability and effectiveness in guiding the identification of active candidate compounds.

### 2.3. Screening Results Based on the Optimal Pharmacophore Model

Leveraging the constructed optimal pharmacophore model, a pharmacophore-guided virtual screening was conducted against a drug-like compound library comprising 50,000 molecules, leading to the initial identification of 1784 potential active candidates. To enhance the reliability of the selection, a Phase Screen Score threshold of ≥2 was applied, yielding a refined subset of 80 high-confidence compounds. The corresponding parameter metrics are detailed in Supplementary Materials Table S2.

### 2.4. PAINS Filtering and ADMET Prediction Results

As summarized in Supplementary Materials Tables S3 and S4, the results of PAINS filtering and ADMET predictions demonstrate that the candidate compounds F1739-0081 and F2571-0016 exhibit favorable profiles across multiple physicochemical and pharmacokinetic parameters. In particular, both compounds show excellent performance in key ADME-related indices, including human intestinal absorption (HIA), oral bioavailability (F_20%_), and blood–brain barrier permeability, suggesting promising in vivo absorption and distribution potential. Toxicity predictions based on the rat oral acute (ROA) model further indicate low predicted toxicity and a favorable safety margin for both candidates. Notably, compound F1739-0081 displays a clearance index (CL) of 9.21, indicative of efficient metabolic elimination, which may confer both desirable metabolic stability and the potential for rapid excretion. Additionally, drug-likeness evaluations presented in Supplementary Materials Table S5 reveal that all selected candidates conform to Lipinski’s Rule of Five, Pfizer’s Rule, and the Golden Triangle criteria, underscoring their favorable pharmacokinetic compatibility and promising drug development potential.

### 2.5. Activity Validation Results

The in vitro antiproliferative activity of the candidate compounds in human lung adenocarcinoma cell lines is detailed in Supplementary Materials Tables S6–S10. All experiments were independently performed in triplicate, encompassing both preliminary screening and subsequent quantitative assessments. The IC_50_ values were calculated using SPSS Statistics 21 software, and statistical analysis of intergroup differences was conducted using two-tailed Student’s *t*-tests, with a significance threshold set at *p* < 0.05. The results demonstrated that (Table 1) compound F1739-0081 exhibited moderate antiproliferative activity in the tested cell lines. Although its inhibitory potency was inferior to that of the positive control Ceritinib, it was slightly superior to that of Lorlatinib, suggesting that F1739-0081 possesses a measurable level of biological activity and may serve as a promising scaffold for further structural optimization and mechanistic investigation. It is further hypothesized that the inhibitory potency of the candidate compound predominantly arises from the preservation of the fused aromatic heterocyclic scaffold and the critical hydrogen bond donor and acceptor groups essential for interaction with the ALK hinge region. Concurrently, the substitution of Ceritinib’s halogenated sulfonamide moiety with a polar aliphatic side chain is anticipated to maintain key hinge-binding interactions while potentially modifying the spatial conformation and interaction profile within the solvent-exposed pocket. This structural alteration may consequently influence the binding mode to the target protein, thereby affecting both binding selectivity and the observed in vitro biological efficacy. In contrast, compound F2571-0016 failed to elicit significant inhibitory effects under identical experimental conditions, with IC_50_ values undeterminable, indicating an absence of discernible antitumor activity within the evaluated concentration range.

### 2.6. Molecular Docking Results

**Figure 3:**
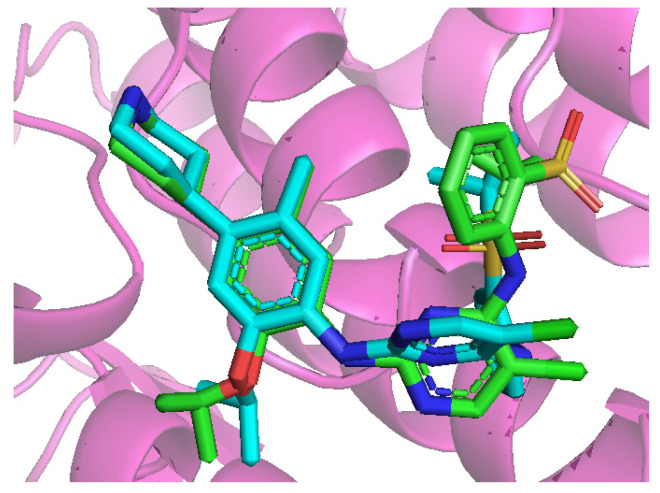
Re-docking results of native ligands. (Green: native ligand (Ceritinib); Blue: re-docked ligand).

As shown in Figure 3, the redocked ligand exhibits a high degree of 3D conformational consistency with the native ligand (Ceritinib), with a RMSD of 1.403 Å (<2 Å). This result indicates that the employed molecular docking protocol is capable of accurately reproducing the binding orientation of known protein–ligand complexes, thereby demonstrating its reliability and suitability for subsequent docking analyses.

**Figure 4:**
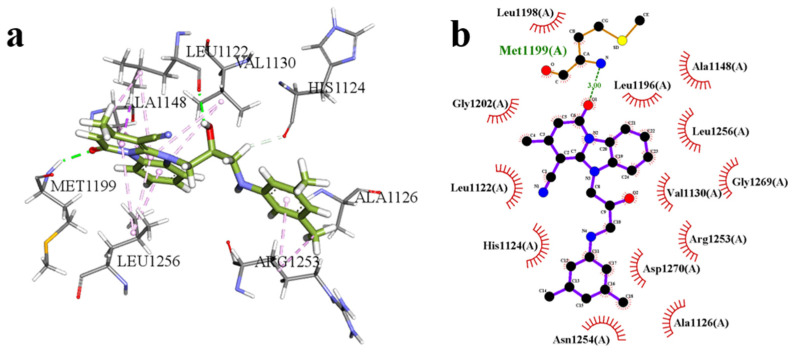
The molecular docking results of the candidate compound F1739-0081; (**a**): 3D interaction diagram, (**b**): 2D interaction diagram.

As illustrated in Figure 4, the candidate compound F1739-0081 adopts a stable conformation within the active site of the target protein, engaging in multiple non-covalent interactions that collectively contribute to its high binding affinity. The carbonyl oxygen and hydroxyl group of the ligand form well-oriented hydrogen bonds with Met1199 and Leu1122, respectively, thereby enhancing the polar complementarity between the ligand and the binding site. The aromatic moiety of the ligand is embedded within a hydrophobic pocket formed by Val1130, Ala1148, and Leu1256, where extensive hydrophobic interactions significantly contribute to the stabilization of the protein–ligand complex. Moreover, additional stabilization and binding specificity are conferred through C–H···π and π–π stacking interactions with His1124 and Arg1253. The docking score for this complex is –8.55 kcal/mol, indicative of a strong and favorable binding interaction.

**Figure 5:**
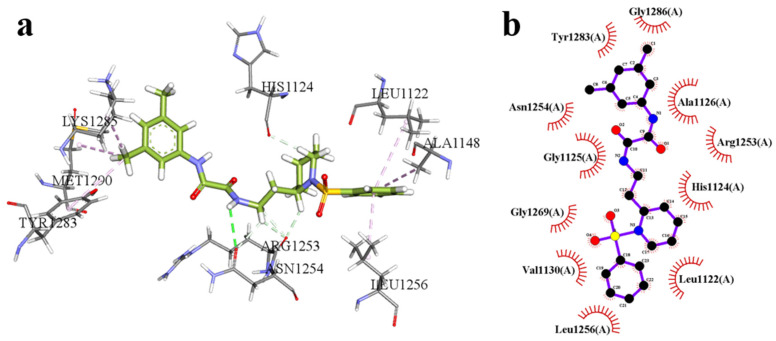
The molecular docking results of the candidate compound F2571-0016; (**a**): 3D interaction diagram, (**b**): 2D interaction diagram.

As illustrated in Figure 5, the candidate compound F2571-0016 is stably accommodated within the active site of the target protein, engaging in a range of key non-covalent interactions that collectively confer high binding affinity. The amide group of the ligand forms a well-oriented hydrogen bond with Asn1254, thereby enhancing intermolecular polar recognition and contributing to binding specificity. Furthermore, the aromatic ring and central rigid scaffold of the ligand establish an extensive hydrophobic interaction network with nonpolar residues including Leu1122, Ala1148, and Leu1256, which significantly stabilizes the binding conformation. π–alkyl interactions are also observed with Tyr1283, Lys1285, and Met1290, further promoting favorable insertion and compatibility within the hydrophobic pocket. In addition, weak C–H···π interactions with His1124 and Arg1253 enhance spatial complementarity and reinforce binding specificity. The composite docking score of –7.95 kcal/mol reflects a strong and energetically favorable binding mode.

### 2.7. Molecular Dynamics Simulation Results

**Figure 6:**
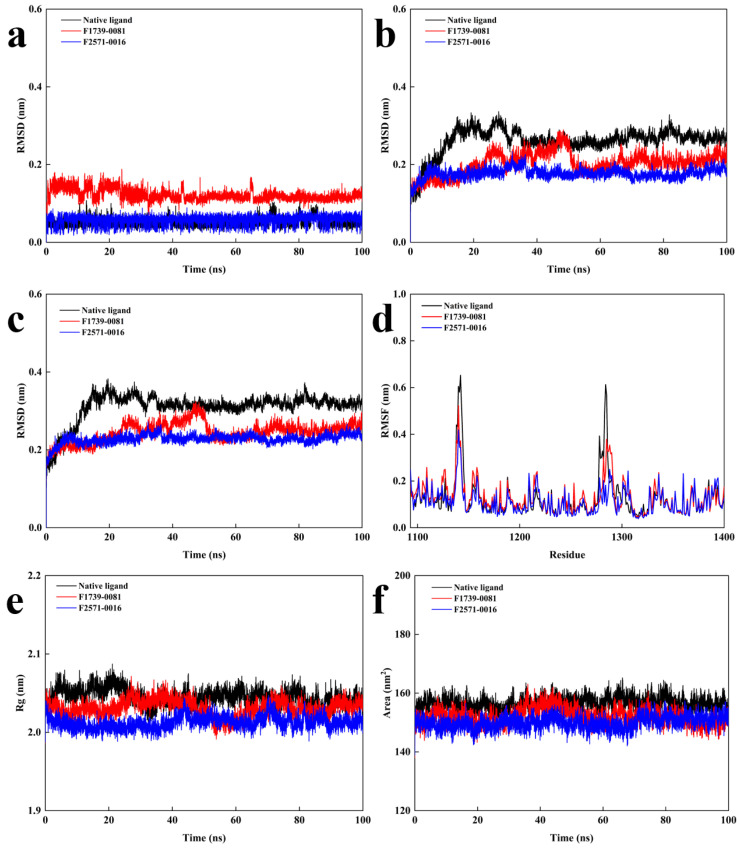
The molecular dynamics simulation results of the candidate compounds; (**a**): RMSD of ligands, (**b**): RMSD of proteins, (**c**): RMSD of complexes, (**d**): RMSF values of complexes, (**e**): Rg of complexes, and (**f**) SASA of complexes.

Figure 6 illustrates the molecular dynamics (MD) simulation results for the candidate compounds. Throughout the 100 ns simulation period, the root-mean-square deviation (RMSD) values of all proteins and protein–ligand complexes, with the exception of the ligand in the F1739-0081 system, remained at relatively low levels, with fluctuations generally confined within 0.1 nm. These values were consistently lower than those observed for the native ligand (Ceritinib) complex, indicating enhanced conformational stability. In particular, the F2571-0016 complex exhibited minimal deviation across the entire simulation timescale, reflecting superior dynamic stability (Figure 6a,c). Root-mean-square fluctuation (RMSF) analysis demonstrated that the residue-level flexibility patterns of the candidate compound–protein complexes closely resembled those of the native ligand (Ceritinib) complex. Notable conformational fluctuations were observed within key structural motifs, including the N-terminal Cα helix, β4–β5 loop, glycine-rich loop (G-loop), and activation loop (A-loop), with particular involvement of residues Leu1122, His1124, and Ala1148 at the distal activation loop region. Additionally, pronounced dynamics were detected near the C-terminal segment of the kinase domain, especially at residues Arg1253 and Leu1256. These results indicate that the candidate compounds modulate the conformational flexibility of critical domains in a manner analogous to the native ligand, supporting their potential binding stability and functional efficacy (Figure 6d). Moreover, the candidate compounds demonstrated consistently lower radius of gyration (Rg) and solvent-accessible surface area (SASA) values relative to the native (Ceritinib) complex, indicating more compact structural conformations with reduced solvent exposure. These observations collectively support the favorable binding compatibility and structural robustness of the candidate compounds (Figure 6e,f).

**Figure 7:**
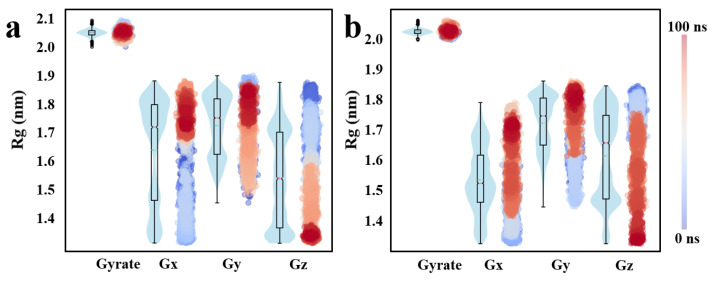
Directional radius of gyration analysis of candidate compounds; (**a**): Compound F1739-0081, (**b**): Compound F2571-0016.

Furthermore, Figure 7 provides a detailed assessment of the Rg and its directional components throughout the molecular dynamic simulation. The results demonstrate that the F2571-0016 complex exhibits reduced fluctuations and a more narrowly distributed profile in both overall Rg values and their directional components compared to F1739-0081, indicative of enhanced conformational stability and structural compactness. In particular, a pronounced and consistent contraction along the Gy axis is observed for F2571-0016, suggesting that the complex adopts a more stable and spatially constrained conformation under simulated physiological conditions.

**Figure 8:**
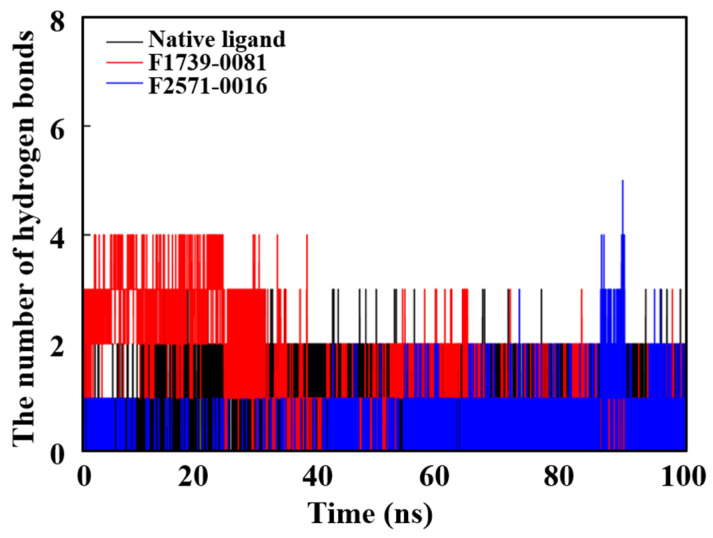
Hydrogen bond interactions of the candidate compounds.

As illustrated in Figure 8, throughout the duration of the molecular dynamic simulation, the candidate compounds consistently sustained between two and three hydrogen bonds with the target protein, exhibiting a stable interaction profile that notably surpasses that of the native ligand (Ceritinib). This robust hydrogen bonding network is instrumental in preserving the structural integrity of the ligand–protein complex, thereby substantially contributing to the enhanced binding affinity of the candidate compounds and the overall conformational stability of the complex.

**Figure 9:**
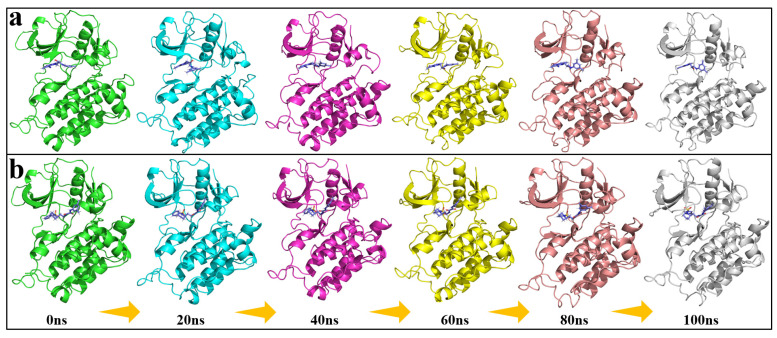
The molecular dynamics simulation snapshots of the candidate compounds; (**a**): Compound F1739-0081, (**b**): Compound F2571-0016.

Furthermore, as depicted in Figure 9, the molecular dynamics simulation snapshots provide a clear depiction of the conformational evolution of the candidate compounds over the course of the simulation. With increasing simulation time, the complexes progressively reached a state of dynamic equilibrium, without evident conformational drift or ligand dissociation, suggesting that the candidate compounds remained stably accommodated within the binding pocket. The binding interface retained a high degree of structural complementarity, with no significant displacement observed at key interaction sites. A stable network of intermolecular interactions between the protein and ligand was maintained, thereby supporting the overall conformational stability of the complexes across the simulation timescale.

**Figure 10:**
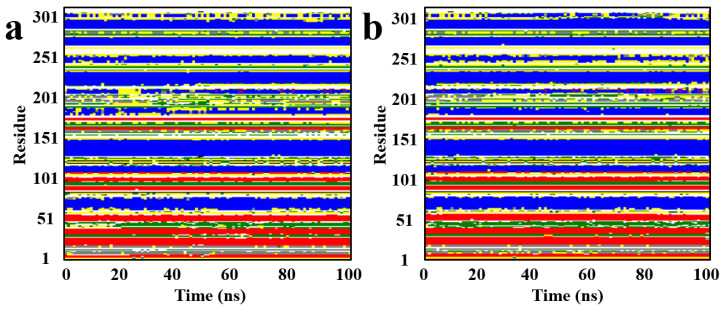
The secondary structure of the protein bound to the candidate compounds; (**a**): Compound F1739-0081, (**b**): Compound F2571-0016; White: Coil; Red: β-Sheet; Black: β-Bridge; Green: Bend; Yellow: Turn; Blue: α-Helix; Purple: 5-Helix; Gray: 3-Helix.

As illustrated in Figure 10, the secondary structure analysis reveals that the proportions of α-helices and β-sheets remained consistently high throughout the simulation. This indicates that the overall architecture of the protein was well preserved and not significantly perturbed by the binding of the candidate compounds. These findings further suggest that the conformational fluctuations of the target protein upon complex formation were minimal, allowing key structural domains to remain intact and the protein’s functional conformation to be maintained over the course of the simulation.

**Figure 11:**
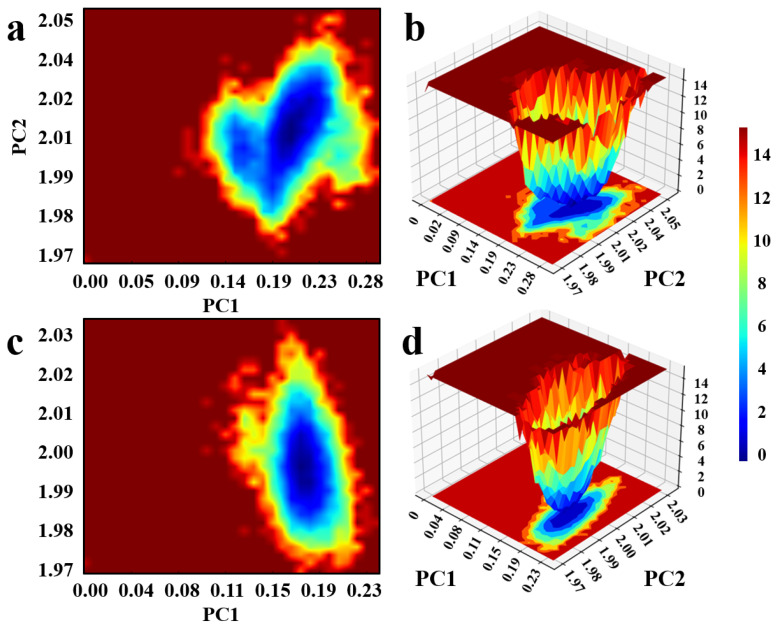
Free energy landscape of candidate compound–protein complexes; (**a**,**b**): Compound F1739-0081, (**c**,**d**): Compound F2571-0016.

Furthermore, as depicted in Figure 11, the 2D and 3D free energy landscapes reveal that the complexes predominantly reside within a single, well-defined energy basin, characterized by a concentrated and continuous low-energy region. The absence of multiple discrete local minima suggests that the system favors a dominant conformational state throughout the simulation, with limited conformational transitions. This indicates that the complexes possess favorable conformational stability and are energetically well stabilized under the simulated conditions.

As presented in Table 2, the binding free energies of the complexes were systematically evaluated using the MM/GBSA approach. Decomposition of the binding free energy components revealed that van der Waals interactions, electrostatic interactions, and gas-phase energies constitute the principal favorable contributors, exerting a predominant influence on complex stabilization. Conversely, polar solvation and total solvation energies displayed antagonistic effects, partially offsetting the favorable interactions and thereby attenuating the overall binding affinity. The candidate compounds exhibited binding free energies of −42.68 kcal/mol and −35.26 kcal/mol, respectively, reflecting a strong thermodynamic propensity for stable binding and corroborating the persistence of a robust binding mode.

**Figure 12:**
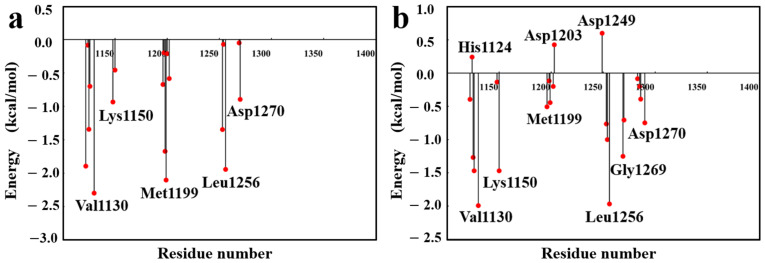
Residue-wise decomposition of binding free energies for the candidate compounds; (**a**): Compound F1739-0081, (**b**): Compound F2571-0016.

The per-residue binding free energy decomposition analysis, as illustrated in Figure 12, reveals that during the binding of F1739-0081 to the target protein, residues Val1130, Lys1150, Met1199, Leu1256, and Asp1270 contribute significantly favorable energetic effects, thereby promoting the stabilization of the complex. Among these, Val1130, Met1199, and Leu1256 exhibit notably pronounced negative binding free energy values, underscoring their pivotal roles in the binding process. Additionally, Lys1150 and Asp1270 provide favorable complementary contributions, collectively sustaining the spatial complementarity and structural integrity of the binding interface. Conversely, the binding of F2571-0016 is predominantly supported by favorable energy contributions from Val1130, Lys1150, Leu1256, and Gly1269, with Val1130 and Leu1256 remaining the primary energetic hotspots, highlighting their conserved and critical functions in ligand recognition. Notably, residues His1124, Asp1203, and Asp1249 display positive binding free energy values in the F2571-0016 complex, suggesting potential electrostatic repulsion or conformational perturbations that may adversely affect the overall binding stability.

## 3. Discussion

This study utilized a structure-based pharmacophore model in conjunction with multi-tiered computational approaches to identify two potential ALK inhibitor candidates. Among these, F1739-0081 demonstrated a measurable degree of antiproliferative activity in vitro, underscoring its viability as a lead compound for further development. Molecular docking and molecular dynamics simulations elucidated its stable binding conformation within the ALK active site and detailed interactions with critical amino acid residues, thereby providing a robust theoretical framework for subsequent structural refinement. Although F2571-0081 exhibited comparatively lower bioactivity, its binding conformation offers valuable insights for rational molecular modification. Notably, the preservation of the fused aromatic heterocyclic scaffold alongside key hydrogen bond donor and acceptor moieties, coupled with modulation of spatial orientation and interaction networks within the solvent-exposed pocket, may enhance binding selectivity and biological efficacy. Furthermore, the integration of PAINS filtering and ADMET prediction substantially improved the pharmacokinetic and safety profiles of the candidate compounds, highlighting the efficacy of computational drug design in early-stage drug discovery. Nevertheless, the current in vitro validation remains preliminary, warranting further comprehensive evaluation across diverse cellular models and in vivo systems to fully characterize the pharmacodynamic properties and safety. Given the clinical challenges posed by ALK resistance mutations, future research should prioritize assessment of candidate compounds’ binding affinity and selectivity toward resistant ALK variants to facilitate their translational advancement.

## 4. Materials and Methods

### 4.1. Database Selection and Ligand Conformation Optimization

The Topscience database (https://www.topscience.cn/) encompasses over 19 million small-molecule compounds, integrating comprehensive physicochemical property data alongside extensive drug-related structural information [21]. This resource is extensively employed in virtual screening and leads compound discovery endeavors. In the present study, a ligand-based pharmacophore conformation-matching approach was utilized to extract a drug-like subset comprising approximately 50,000 compounds from the database for virtual screening. This strategy aimed to efficiently identify candidate molecules with potential ALK inhibitory activity and favorable drug development profiles, thereby providing a robust theoretical foundation for subsequent structural optimization and bioactivity validation.

Prior to virtual screening, five clinically approved ALK inhibitors were selected to serve as the foundation for the pharmacophore model construction. These included representative compounds from the first generation (Crizotinib), second generation (Alectinib, Ceritinib, Brigatinib), and third generation (Lorlatinib), thereby ensuring that the model encompassed critical structural features and mechanisms of action characteristics of inhibitors across different generations. The co-crystal structures utilized in this study comprise PDB IDs 2YFX, 3AOX, 4MKC, 6MX8, and 4CLI. Notably, the 2YFX structure contains the L1196M resistance mutation, whereas the 3AOX, 4MKC, 6MX8, and 4CLI structures correspond to wild-type ALK kinase complexes. The inclusion of these structures provides a robust structural basis for the construction of the pharmacophore model. To ensure chemical validity and spatial consistency of the input conformations, the selected ligands underwent systematic preprocessing, which primarily involved 3D conformation optimization and chemical structure standardization [22,23]. Additionally, to address ligand conformational flexibility, multiple low-energy conformers were generated for each molecule using the ConfGen algorithm [24], providing diverse candidate inputs for pharmacophore modeling.

### 4.2. Pharmacophore Modeling

The ATP binding pocket of ALK kinase was employed as the conformational reference framework. Five representative inhibitors were structurally aligned and superimposed according to their binding modes observed in protein–ligand co-crystal complexes, ensuring spatial congruence and precise alignment of key pharmacophoric features. Subsequently, a systematic analysis of the inhibitors’ interaction patterns within the ATP binding site was performed, with particular emphasis on stable hydrogen bond interactions involving conserved residues in the hinge region. Shared spatial configurations and functional group characteristics were extracted to construct a representative pharmacophore model comprehensively capturing the critical pharmacophoric features required for effective ALK inhibitor engagement. The pharmacophore model was developed utilizing the “Develop Pharmacophore Model” function in the Phase module of Schrödinger 12.8. Specific parameters included retention of all known active ligands’ 3D conformations, alignment based on their spatial arrangement within the binding pocket, inclusion of hydrogen bond donors (HBD), hydrogen bond acceptors (HBA), hydrophobic centers (HY), and aromatic rings (AR) as candidate pharmacophoric features, a tolerance radius of 1.2 Å to ensure spatial precision in feature matching, and activation of the Exclusion Volume feature to simulate steric constraints of the binding site, thereby enhancing the model’s selectivity and accuracy in subsequent virtual screening applications.

### 4.3. Pharmacophore Model Validation

Prior to the application of the optimal pharmacophore model in subsequent virtual screening, a systematic evaluation of its discriminative capability is imperative to validate its effectiveness in differentiating active compounds from inactive ones. The model’s performance is quantitatively assessed via the Receiver Operating Characteristic (ROC) curve [25], which provides a visual representation of the trade-off between sensitivity and specificity [26]. The Area Under the Curve (AUC) serves as a key metric reflecting the overall discriminative power of the model [27]; values approaching unity denote superior accuracy in distinguishing active from inactive molecules [28]. Furthermore, a ROC curve proximate to the upper left corner indicates a model that achieves both high sensitivity and low false-positive rates, thereby demonstrating excellent predictive performance.

### 4.4. PAINS Filtering and ADMET Prediction

To improve the reliability of virtual screening outcomes and minimize the potential risk of false positives during subsequent bioactivity validation, candidate compounds identified from pharmacophore-based screening were initially subjected to Pan Assay Interference Compounds (PAINS) structural alert filtering [29]. This step aimed to eliminate molecules prone to non-specific biological interference, thereby ensuring the structural integrity and target specificity of the selected candidates. Upon completion of PAINS filtering, an in silico evaluation of pharmacokinetic and drug-likeness profiles was performed based on ADMET (Absorption, Distribution, Metabolism, Excretion, and Toxicity) parameters [30]. The objective was to further refine the candidate pool by selecting compounds exhibiting favorable ADMET characteristics and low toxicity potential, thus providing a robust theoretical basis for subsequent experimental validation.

### 4.5. Molecular Docking

To systematically evaluate the binding conformations of the screened candidate compounds with the target protein and to assess their potential as ALK inhibitors, molecular redocking of the co-crystallized ligand was first conducted. The root-mean-square deviation (RMSD) between the experimentally determined conformation and the redocked pose was employed as a key metric to validate the accuracy and reliability of the docking protocol. To ensure structural accuracy and clinical relevance in subsequent molecular docking studies, a systematic comparative analysis was performed on five resolved ALK crystal structures [31,32,33,34]. Taking into account key factors such as structural resolution, the clinical approval status of co-crystallized ligands, and the presence of clinically significant mutations, PDB ID: 4MKC was ultimately selected as the receptor structure for docking investigations. This structure offers a high resolution of 2.01 Å and features Ceritinib, an FDA-approved second-generation ALK inhibitor, as the co-crystallized ligand [33]. This is particularly favorable for establishing a reliable model in preliminary molecular docking studies.

Subsequently, molecular docking studies were carried out using AutoDock Vina v1.2.3 to investigate the binding interactions between the candidate compounds and the ALK protein. Prior to docking, both ligands and the receptor were subjected to geometric optimization, and crystallographic water molecules not directly involved in ligand binding were removed to enhance the accuracy of the simulations [35]. Ligand optimization was conducted using the Tripos force field with a non-bonded interaction cutoff of 9.0 Å and a dielectric constant of 2 [36], while the protein was prepared using the Kollman force field under the same non-bonded cutoff and a dielectric constant of 4 [37]. The docking grid box was defined with dimensions of X = 45 Å, Y = 45 Å, and Z = 45 Å, a grid spacing of 0.375 Å, and centered at X = −20.12, Y = 9.02, Z = −6.55, ensuring comprehensive coverage of the active site region. The resulting ligand–protein complexes were subsequently visualized and analyzed using Discovery Studio Visualizer 2019 to systematically elucidate potential binding modes and key intermolecular interactions.

### 4.6. Activity Validation

To quantitatively assess the in vitro inhibitory effects of the candidate compounds on the proliferation of human lung adenocarcinoma A549 cells, an MTT assay was employed. A549 cells were retrieved from cryopreservation and cultured under standard conditions until reaching the logarithmic growth phase prior to experimentation. Subsequently, cells exhibiting optimal viability were seeded into 96-well plates at a density of 4 × 10^3^ cells per well and incubated overnight at 37 °C in a humidified atmosphere containing 5% CO_2_.

Following 24 h of adherence, the culture medium was aspirated, and cells were gently washed twice with phosphate-buffered saline (PBS). Cells were then exposed to gradient concentrations of the candidate compounds across five levels, with each concentration tested in triplicate. Ceritinib and Lorlatinib served as the positive control, while untreated cells constituted the negative control. After 48 h of treatment, 10 μL of MTT reagent was added to each well, and incubation proceeded for an additional 4 h. Thereafter, 100 μL of formazan solubilization solution was introduced, and the plates were thoroughly mixed to ensure complete dissolution of formazan crystals. Absorbance values were measured at 570 nm using a microplate reader, enabling calculation of relative cell viability. The half-maximal inhibitory concentration (IC_50_) values were subsequently derived from dose–response curves to quantitatively evaluate the antiproliferative potency of the candidate compounds against A549 cells.

### 4.7. Molecular Dynamics Simulation

To elucidate the potential mechanisms of action of the candidate compounds and evaluate the conformational stability of their complexes with the target protein under near-physiological conditions, molecular dynamics simulations were conducted using GROMACS version 2022.3 [38]. The simulation system was established under the isothermal–isobaric (NPT) ensemble, maintaining a temperature of 300 K and a pressure of 1 bar [39]. The Amber99sb-ildn force field was employed for system parameterization, while solvation was modeled using the TIP3P water model [40]. To ensure electro-neutrality of the system, an appropriate number of Na^+^ and Cl^−^ ions were incorporated [39].

Prior to production simulations, the system underwent energy minimization employing a combination of the steepest descent and conjugate gradient methods to effectively eliminate unfavorable steric clashes and enhance conformational stability [41]. Subsequently, the system was equilibrated under the canonical ensemble (NVT) for 100,000 steps to achieve thermal stabilization, followed by a 100 ps equilibration under the NPT with a coupling time constant of 0.1 ps to ensure pressure equilibration [42]. Upon completion of equilibration, a 100 ns molecular dynamics production run was performed. The resulting trajectories were systematically analyzed using the integrated GROMACS 2022.3 analysis tools to elucidate the dynamic behavior and conformational stability of the candidate compounds within the target protein complexes, thereby providing a robust theoretical framework for mechanistic interpretation.

## 5. Conclusions

In this study, a structure-based pharmacophore model was developed using the three-dimensional conformations of five approved ALK small-molecule inhibitors. Integrating PAINS filtering, ADMET profiling, and molecular docking techniques, a systematic virtual screening of the Topscience compound library was performed, leading to the identification of two candidate compounds with potential bioactivity: F1739-0081 and F2571-0016. In vitro MTT assay results revealed that the candidate compound F1739-0081 exerted measurable antiproliferative effects against the A549 cell line, with an IC_50_ value of 261.7 μM. Although its inhibitory potency is inferior to that of the positive control Ceritinib, it demonstrates a slight advantage over Lorlatinib, thereby indicating a favorable competitive profile. The introduced structural modifications confer enhanced spatial adaptability and interaction versatility to F1739-0081, underscoring its significant potential for further optimization. It is therefore envisaged that rational structural modifications could further improve the compound’s binding affinity and selectivity toward the target, ultimately augmenting its pharmacodynamic efficacy in both in vitro and in vivo settings. In contrast, F2571-0016 demonstrated no appreciable inhibitory effect. Molecular docking analyses revealed that both F1739-0081 and F2571-0016 could stably occupy the ALK active site, with docking scores of –8.55 kcal/mol and −7.95 kcal/mol, respectively. Subsequent molecular dynamics simulations confirmed the conformational stability of the ligand–protein complexes, as reflected by low RMSD and RMSF fluctuations. MM/GBSA-based binding free energy calculations yielded values of −42.68 kcal/mol and −35.26 kcal/mol, respectively, supporting strong binding affinity. Energy decomposition analyses further identified key residues, including Val1130, Met1199, Leu1256, and Asp1270, as major contributors to complex stabilization. Overall, this work integrates structure-based pharmacophore modeling with multi-level computational and experimental approaches to evaluate the potential of F1739-0081 and F2571-0016 as ALK inhibitors, offering valuable insights for future structure-based drug design and development targeting ALK.

## Figures and Tables

**Table 1:** The biological activities of candidate compounds.

NO	IC_50_ Values (μM)
	A549
F1739-0081	261.7
F2571-0016	>500
Ceritinib	33.85
Lorlatinib	265.6

**Table 2:** Free energy of candidate compounds’ complex binding.

Energy Component	Native Ligand (kcal/mol)	F1739-0081 (kcal/mol)	F2571-0016 (kcal/mol)
Δ*E_vdW_*	−61.25	−52.01	−50.50
Δ*E_ele_*	−29.06	−6.80	−15.55
Δ*E_polar_*	50.82	22.36	37.66
Δ*E_nonpolar_*	−7.56	−6.23	−6.87
Δ*G_gas_*	−90.31	−58.81	−66.05
Δ*G_solv_*	43.26	16.13	30.80
Δ*G_MMGBSA_*	−47.05	−42.68	−35.26

## Data Availability

Data are contained within the article or Supplementary Materials.

## Supplementary Materials

The following supporting information can be downloaded at: https://www.mdpi.com/article/10.3390/ph18081207/s1. Table S1. Pharmacophore modeling results. Table S2. Pharmacophore screening results. (TOP 80). Table S3. PAINS filtering and drug-likeness prediction. Table S4. Prediction of ADMET properties of candidate compounds. Table S5. Drug similarity and synthetic feasibility of candidate compounds. Table S6. Blank and control. Table S7. Compound F1739-0081 (A549). Table S8. Compound F2571-0016 (A549). Table S9. Ceritinib (A549). Table S10. Lorlatinib (A549).
