# Supplementary material for: Multiscale Computational and Pharmacophore-Based Screening of ALK Inhibitors with Experimental Validation
Source: Pharmaceuticals (Basel). 2025 Aug 15;18(8):1207. doi: 10.3390/ph18081207 (PMC12389212; doi:10.3390/ph18081207)
Supplement: Supplementary file 1 [file pharmaceuticals-18-01207-s001.zip › pharmaceuticals-3803148-supplementary.pdf]

Multiscale Computational and Pharmacophore-Based Screening of ALK Inhibitors with Experimental Validation

Ya-Kun Zhang [1], Jian-Bo Tong [1,*], Yue Sun [1] and Yan-Rong Zeng [2,*]

1 *College of Chemistry and Chemical Engineering, Shaanxi University of Science and Technology, Xi'an 710021, China*

2 *School of Chinese Ethnic Medicine, Guizhou Minzu University, Guiyang 550025, China*

* *Correspondence: jianbotong@sust.edu.cn (J.-B.T.); yrong1992@163.com (Y.-R.Z.)*

1. Pharmacophore modeling

Table S1. Pharmacophore modeling results.

Compound	Stars	Volume Score	Fitness	PhaseScreenScore
Ceritinib	3	0.559	2.326	2.326
Brigatinib	3	0.454	1.832	1.832
Crizotinib	3	0.393	1.419	1.419
Alectinib	3	0.342	1.322	1.322
Lorlatinib	3	0.222	0.892	0.892

Table S2. Pharmacophore screening results. (TOP 80)

Compound	PhaseScreenScore	SMILES
F3371-0804	2.211	Cc1ccc(-c2c(CNS(=O)(=O)c3ccccc3)c(=O)[nH]c3ccccc23)cc1
F1739-0078	2.188	Cc1cc(=O)n2c3ccccc3n(C[C@H](O)CNc3ccc(F)cc3)c2c1C#N
F1739-0081	2.176	Cc1cc(C)cc(NC[C@H](O)Cn2c3ccccc3n3c(=O)cc(C)c(C#N)c23)c1
F3273-0814	2.157	N#C/C(C([NH-])=O)=C1/S[C@H](Cc2cccc([N+](=O)[O-])c2)C(=O)N1c1ccccc1
F0324-0022	2.145	CC(=O)N1N=C(c2c(-c3ccccc3)c3cc(Cl)ccc3[nH]c2=O)C[C@@H]1c1ccccc1Cl

F1881-0550	2.136	CC(=[OH+])/N=c1\nc(SCC(N)=O)[nH]c([O-])c1NC(=O)c1ccc(F)cc1
F0324-0022	2.125	CC(=O)N1N=C(c2c(-c3ccccc3)c3cc(Cl)ccc3[nH]c2=O)C[C@H]1c1ccccc1Cl
F3273-0814	2.105	N#C/C(C(N)=O)=C1/S[C@@H](Cc2cccc([N+](=O)[O-])c2)C(=O)N1c1ccccc1
F5112-0038	2.105	Cc1ccc(S(=O)(=O)c2nc(-c3ccccc3)[nH]c2SCC(N)=O)cc1
F6524-1336	2.102	CC(C)(C)CC(=O)NCc1nc(-c2cc[nH]c(=O)c2)no1
F3273-0814	2.099	N#C/C(C(N)=O)=C1/S[C@H](Cc2cccc([N+](=O)[O-])c2)C(=O)N1c1ccccc1
F3273-0814	2.098	N#C/C(C([NH-])=O)=C1/S[C@@H](Cc2cccc([N+](=O)[O-])c2)C(=O)N1c1ccccc1
F3371-0659	2.095	COCC(=O)N1N=C(c2c(-c3ccccc3)c3ccccc3[nH]c2=O)C[C@H]1c1ccc(C)cc1
F5112-0038	2.095	Cc1ccc(S(=O)(=O)c2[nH]c(-c3ccccc3)nc2SCC(N)=O)cc1
F1739-0081	2.093	Cc1cc(C)cc(NC[C@@H](O)Cn2c3ccccc3n3c(=O)cc(C)c(C#N)c23)c1
F1057-0410	2.09	O=C1N[C@@H](c2ccc3c(c2)OCO3)[C@@H](C(=O)c2ccccc2)[C@@](O)(C(F)(F)F)N1
F0770-0062	2.089	O=C([O-])CCCC(=O)N1N=C(c2c(-c3ccccc3)c3cc(Cl)ccc3[nH]c2=O)C[C@H]1c1ccc(Br)cc1
F2571-0016	2.077	Cc1cc(C)cc(NC(=O)C(=O)NCC[C@H]2CCCCN2S(=O)(=O)c2ccccc2)c1
F3358-0053	2.064	N#Cc1c(C(F)(F)F)cc(-c2ccco2)nc1SCC(N)=O
F1739-0089	2.063	Cc1cc(=O)n2c3ccccc3n(C[C@H](O)CNc3ccc(C(C)C)cc3)c2c1C#N

F1425-0428	2.062	CCCN1C(=O)/C(=C/c2c(N3CCNC(=O)[C@H]3CC(=O)OC)nc3c(C)cccn3c2=O)SC1=S
F0682-0795	2.062	NC(=O)CSc1ncnc2c1nnn2-c1ccc(F)cc1
F2359-0574	2.062	Cc1cc(Cl)ccc1Nc1nc(Nc2ccc(F)cc2)nc2nccnc12
F0454-0008	2.061	N#Cc1c(N)nc(SCC(N)=O)c(C#N)c1-c1ccc(Cl)cc1
F2571-0057	2.061	COc1ccccc1CNC(=O)C(=O)NCC[C@@H]1CCCCN1S(=O)(=O)c1ccccc1
F3250-0089	2.056	Cc1cc(=O)n2c3ccccc3n(C[C@@H](O)CNCc3ccccc3)c2c1C#N
F2571-0009	2.053	Cc1ccc(NC(=O)C(=O)NCC[C@H]2CCCCN2S(=O)(=O)c2ccccc2)cc1
F5882-3041	2.051	O=C1CN(c2ccccc2NC(=O)NCc2cccc3ccccc23)CCN1
F3184-0160	2.051	[H]/N=C1/NC(=O)S/C1=C\c1cc(Cl)ccc1OCC(=O)Nc1ccc(Cl)cc1
F6756-1068	2.051	N#Cc1cc2c(nc1N1CC[C@](F)(C(N)=O)C1)CCOC2
F2571-0358	2.05	COc1ccc(NC(=O)C(=O)NCC[C@H]2CCCCN2S(=O)(=O)c2cccs2)cc1
F1492-0011	2.048	CS(=O)(=O)N1N=C(c2c(-c3ccccc3)c3cc(Cl)ccc3[nH]c2=O)C[C@H]1c1cccs1
F5736-0030	2.047	Cc1nc(NCC[NH+]2CCOCC2)nc(Nc2ccccc2)c1[N+](=O)[O-]
F1190-0258	2.044	CCOC(=O)c1c(C(C)(C)C)oc2ccc(OCC(N)=O)cc12
F6524-1360	2.042	O=C(NCc1nc(-c2cc[nH]c(=O)c2)no1)c1ccc(F)cc1Cl

F6524-1321	2.04	Cn1cccc(C(=O)NCc2nc(-c3cc[nH]c(=O)c3)no2)c1=O
F2678-0503	2.037	COc1ccc(OC)c(Nc2nc(NCC[NH+]3CCOCC3)nc(N)c2[N+](=O)[O-])c1
F2571-0403	2.034	COc1ccccc1CNC(=O)C(=O)NCC[C@H]1CCCCN1S(=O)(=O)c1cccs1
F1492-0011	2.033	CS(=O)(=O)N1N=C(c2c(-c3ccccc3)c3cc(Cl)ccc3[nH]c2=O)C[C@@H]1c1cccs1
F1739-0085	2.032	Cc1cc(=O)n2c3ccccc3n(C[C@H](O)CN3CC[NH+](Cc4ccc(Cl)cc4)CC3)c2c1C#N
F6524-1331	2.031	Cc1noc(C)c1CCC(=O)NCc1nc(-c2cc[nH]c(=O)c2)no1
F6524-1365	2.03	O=C(NCc1nc(-c2cc[nH]c(=O)c2)no1)c1nc2ccccc2s1
F6548-2260	2.029	CCc1ccc(C(=O)c2cnc3ccc(OC)cc3c2N2CCC(C(N)=O)CC2)cc1
F6524-1384	2.029	O=C(NCc1nc(-c2cc[nH]c(=O)c2)no1)c1ccc2[nH]ccc2c1
F6524-1319	2.029	O=C(NCc1nc(-c2cc[nH]c(=O)c2)no1)c1cc2ccccc2[nH]1
F6524-1328	2.028	O=C(NCc1nc(-c2cc[nH]c(=O)c2)no1)c1ccc2[nH+]c[nH]c2c1
F6524-1328	2.028	O=C(NCc1nc(-c2cc[nH]c(=O)c2)no1)c1ccc2nc[nH]c2c1
F6524-1328	2.028	O=C(NCc1nc(-c2cc[nH]c(=O)c2)no1)c1ccc2[nH]cnc2c1
F5736-0030	2.028	Cc1nc(NCCN2CCOCC2)nc(Nc2ccccc2)c1[N+](=O)[O-]
F6190-1830	2.027	O=C(NCC[C@@H](O)c1cccs1)C(=O)Nc1cccnc1

F6524-1353	2.027	O=C(CCCc1ccccc1)NCc1nc(-c2cc[nH]c(=O)c2)no1
F6524-1369	2.022	O=C(NCc1nc(-c2cc[nH]c(=O)c2)no1)c1cnc2ccccc2n1
F1739-0074	2.022	Cc1ccc(NC[C@@H](O)Cn2c3ccccc3n3c(=O)cc(C)c(C#N)c23)cc1C
F6521-7436	2.021	Cn1ccc2cc([C@H](O)CNC(=O)N3CCNC3=O)ccc21
F3385-3012	2.021	CCOC(=O)c1c(-c2ccc(OC)cc2)oc2cc(Br)c(OCC(N)=O)cc12
F2571-0466	2.017	O=C(NCC[C@H]1CCCCN1S(=O)(=O)c1ccc(Cl)cc1)C(=O)NCc1ccco1
F2574-0535	2.017	Cc1ccc(C)c(S(=O)(=O)N2CCC[C@@H]2CNC(=O)C(=O)Nc2cccc(Cl)c2)c1
F3280-0040	2.015	CS(=O)(=O)[N-]c1ccccc1C(=O)C[C@]1(O)C(=O)Nc2ccc(Cl)cc21
F0016-1324	2.015	O=C(/C=C/c1ccco1)c1c(-c2ccccc2)c2cc(Br)ccc2[nH]c1=O
F0216-0036	2.013	O=C([O-])CCC(=O)N1N=C(c2c(-c3ccccc3)c3cc(Cl)ccc3[nH]c2=O)C[C@@H]1c1ccccc1
F2571-0105	2.012	COc1ccc(CNC(=O)C(=O)NCC[C@@H]2CCCCN2S(=O)(=O)c2ccc(F)cc2)cc1
F2571-0042	2.011	O=C(NCC[C@H]1CCCCN1S(=O)(=O)c1ccccc1)C(=O)NCc1cccnc1
F5481-1940	2.01	Cc1ccc(-n2ncc3c(C)nnc(SCC(N)=O)c32)cc1
F3406-4590	2.01	O=C(CSc1nc2ccccc2c(=O)n1C[C@H]1CCCO1)/N=c1\[nH]cn[nH]1
F1366-2960	2.01	CCOC(=O)Cn1/c(=N\C(=O)c2cc(-c3cccs3)[nH+]c3ccccc23)sc2cc(S(N)(=O)=O)ccc21

F2571-0138	2.01	O=C(NCC[C@H]1CCCCN1S(=O)(=O)c1ccc(F)cc1)C(=O)NCc1ccccc1Cl
F2571-0458	2.009	O=C(NCC[C@H]1CCCCN1S(=O)(=O)c1ccc(Cl)cc1)C(=O)NCc1cccnc1
F2359-0707	2.009	c1ccc(Nc2nc(NCC[NH+]3CCOCC3)nc3nccnc23)cc1
F2571-0487	2.007	O=C(NCC[C@H]1CCCCN1S(=O)(=O)c1ccc(Cl)cc1)C(=O)NCc1ccccc1F
F3371-0659	2.006	COCC(=O)N1N=C(c2c(-c3ccccc3)c3ccccc3[nH]c2=O)C[C@@H]1c1ccc(C)cc1
F1425-0428	2.004	CCCN1C(=O)/C(=C/c2c(N3CCNC(=O)[C@@H]3CC(=O)OC)nc3c(C)cccn3c2=O)SC1=S
F5481-2101	2.003	Cc1ccc(-n2ncc3c(C(C)C)nnc(SCC(N)=O)c32)cc1
F1619-0719	2.003	O=C(COc1ccc(Cl)cc1/C=C1\SC(=O)NC1=O)Nc1ccccc1
F2002-0419	2.002	COc1ccc(S(=O)(=O)N2CCCO[C@H]2CNC(=O)C(=O)NCCC[NH+]2CCOCC2)cc1C
F1739-0074	2.002	Cc1ccc(NC[C@H](O)Cn2c3ccccc3n3c(=O)cc(C)c(C#N)c23)cc1C
F0770-0062	2.002	O=C([O-])CCCC(=O)N1N=C(c2c(-c3ccccc3)c3cc(Cl)ccc3[nH]c2=O)C[C@@H]1c1ccc(Br)cc1
F3385-1508	2.001	C=C(C)COc1ccc(-c2nc(N)nc(C)c2Oc2ccccc2OC)c(O)c1
F6524-1467	2.001	O=c1cc(-c2noc(CNS(=O)(=O)c3ccc4c(c3)CCO4)n2)cc[nH]1
F6190-1871	2.001	O=C(NCC[C@@H](O)c1cccs1)C(=O)Nc1ccc2c(c1)OCO2
F1739-0091	2	CCc1ccc(NC[C@H](O)Cn2c3ccccc3n3c(=O)cc(C)c(C#N)c23)cc1

2. Drug-likeness prediction

Table S3. PAINS filtering and drug-likeness prediction.

Compound	PAINS	TPSA	H-bond acceptors	H-bond donors	Rotatable bonds	MR	MW	Synthetic Accessibility	Leadlikeness
F3371-0804	0	87.41	4	2	5	115.35	404.48	3.22	2
F1739-0078	0	82.46	4	2	5	109.67	390.41	3.42	1
F1739-0081	0	82.46	3	2	5	119.64	400.47	3.63	1
F3273-0814	0	144.32	6	1	5	109.34	393.4	4.02	1
F0324-0022	0	65.53	3	1	4	140.61	476.35	4.03	2
F1881-0550	0	181.82	7	4	7	89.87	379.37	3.24	1
F0324-0022	0	65.53	3	1	4	140.61	476.35	4.03	2
F3273-0814	0	158.31	5	1	5	108.79	394.4	4.05	1
F5112-0038	0	139.59	4	2	6	100.36	387.48	3.35	1
F6524-1336	0	100.88	5	2	6	76.9	290.32	2.93	0
F3273-0814	0	158.31	5	1	5	108.79	394.4	4.05	1
F3273-0814	0	144.32	6	1	5	109.34	393.4	4.02	1

F3371-0659	0	74.76	4	1	6	141.45	451.52	4.22	2
F5112-0038	0	139.59	4	2	6	100.36	387.48	3.35	1
F1739-0081	0	82.46	3	2	5	119.64	400.47	3.63	1
F1057-0410	0	96.89	8	3	4	99.71	408.33	3.79	1
F0770-0062	0	105.66	5	1	8	157.55	591.86	4.29	3
F2571-0016	0	103.96	5	2	9	124.65	443.56	3.7	3
F3358-0053	0	118.21	7	1	5	71.09	327.28	3.01	0
F1739-0089	0	82.46	3	2	6	124.29	414.5	3.72	1
F1425-0428	1	170.71	6	1	7	148.1	515.61	4.54	1
F0682-0795	0	124.88	6	1	4	73.85	304.3	2.58	0
F2359-0574	0	75.62	5	2	4	104.15	380.81	3.05	2
F0454-0008	0	154.88	4	2	4	87.95	343.79	2.83	0
F2571-0057	0	113.19	6	2	11	124.48	459.56	3.68	2
F3250-0089	0	82.46	4	2	6	112.98	386.45	3.47	1
F2571-0009	0	103.96	5	2	9	119.68	429.53	3.56	2

F5882-3041	0	73.47	2	3	6	117.5	374.44	2.78	1
F3184-0160	0	116.58	4	3	6	112.87	422.29	3.31	2
F6756-1068	0	92.24	5	1	2	75.11	290.29	3.26	0
F2571-0358	0	141.43	6	2	10	119.09	451.56	3.83	2
F1492-0011	0	119.22	4	1	4	137.44	483.99	4.17	2
F5736-0030	0	109.33	5	3	7	105.45	359.4	3.44	1
F1190-0258	0	91.76	5	1	7	85.78	319.35	3.26	0
F6524-1360	0	100.88	6	2	5	82.81	348.72	2.75	0
F6524-1321	0	122.88	6	2	5	83.36	327.29	2.86	0
F2678-0503	0	153.81	7	4	9	117.87	420.44	3.79	2
F2571-0403	0	141.43	6	2	11	122.36	465.59	3.88	2
F1492-0011	0	119.22	4	1	4	137.44	483.99	4.17	2
F1739-0085	0	78.11	4	2	6	146.88	491	4.26	1
F6524-1331	0	126.91	7	2	7	87.22	343.34	3.37	0
F6524-1365	0	142.01	6	2	5	91.02	353.36	3.04	1

F6548-2260	0	85.52	4	1	6	125.05	417.5	2.99	2
F6524-1384	0	116.67	5	3	5	89.69	335.32	2.82	0
F6524-1319	0	116.67	5	3	5	89.69	335.32	2.84	0
F6524-1328	0	130.81	5	4	5	88.38	337.31	2.83	0
F6524-1328	0	129.56	6	3	5	87.49	336.3	2.8	0
F6524-1328	0	129.56	6	3	5	87.49	336.3	2.8	0
F5736-0030	0	108.13	6	2	7	104.49	358.39	3.4	1
F6190-1830	0	119.56	4	3	8	79.5	305.35	2.92	1
F6524-1353	0	100.88	5	2	8	92.04	338.36	2.95	1
F6524-1369	0	126.66	7	2	5	90.93	348.32	2.94	0
F1739-0074	0	82.46	3	2	5	119.64	400.47	3.63	1
F6521-7436	0	86.6	3	3	5	88.76	302.33	2.82	0
F3385-3012	0	100.99	6	1	8	106.13	448.26	3.57	3
F2571-0466	0	117.1	6	2	10	115.26	453.94	3.74	2
F2574-0535	0	103.96	5	2	8	120.05	449.95	3.59	3

F3280-0040	0	108.92	6	2	5	100.16	393.82	3.01	1
F0016-1324	0	63.07	3	1	4	109.78	420.26	3.19	2
F0216-0036	0	105.66	5	1	7	145.05	498.94	4.18	2
F2571-0105	0	113.19	7	2	11	124.44	477.55	3.7	2
F2571-0042	0	116.85	6	2	10	115.78	430.52	3.52	2
F5481-1940	0	111.99	4	1	4	86.03	313.38	2.8	0
F3406-4590	0	143.32	6	2	6	99.64	386.43	3.81	1
F1366-2960	0	199.82	7	2	8	143.62	553.65	4.18	3
F2571-0138	0	103.96	6	2	10	122.95	481.97	3.68	3
F2571-0458	0	116.85	6	2	10	120.79	464.97	3.57	2
F2359-0707	0	89.29	5	3	6	104.76	352.41	3.33	1
F2571-0487	0	103.96	6	2	10	122.95	481.97	3.69	3
F3371-0659	0	74.76	4	1	6	141.45	451.52	4.22	2
F1425-0428	1	170.71	6	1	7	148.1	515.61	4.54	1
F5481-2101	0	111.99	4	1	5	95.64	341.43	3.05	0

F1619-0719	0	109.8	4	2	6	104.47	388.82	3.27		2
F2002-0419	0	136.09	8	3	12	132.02	499.6	4.58		2
F1739-0074	0	82.46	3	2	5	119.64	400.47	3.63		1
F0770-0062	0	105.66	5	1	8	157.55	591.86	4.29		3
F3385-1508	0	99.72	6	2	7	112.31	393.44	3.52		2
F6524-1467	0	135.56	8	2	5	90.08	374.37	3.14		1
F6190-1871	0	125.13	5	3	8	87.77	348.37	3.23		1
F1739-0091	0	82.46	3	2	6	119.48	400.47	3.61		1

3. Molecular docking

Molecular docking results.

mol	rseq	mseq	S	rmsd_refine
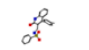	1	1	-5.4676	2.8903
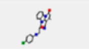	1	2	-5.6080	1.5160
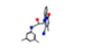	1	3	-5.4928	2.8455
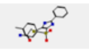	1	4	-5.4315	1.0568
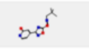	1	5	-5.0215	1.3950
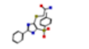	1	6	-6.0297	2.3849
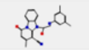	1	7	-6.0549	1.5896
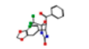	1	8	-4.9046	1.4257
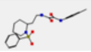	1	9	-6.3062	1.2015
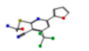	1	10	-4.5747	1.6254
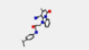	1	11	-5.7255	2.4691
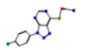	1	12	-5.0484	1.0727
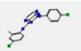	1	13	-5.5523	3.6408
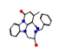	1	14	-5.5095	1.3660
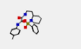	1	15	-5.7350	1.6244
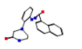	1	16	-5.7043	3.9879
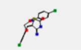	1	17	-5.4215	2.5068
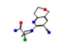	1	18	-4.9480	1.6117
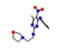	1	19	-5.2589	1.5938
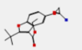	1	20	-5.8361	0.8783
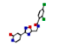	1	21	-5.3487	1.9909
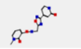	1	22	-5.3544	1.0990
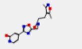	1	23	-5.9356	1.1881
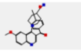	1	24	-5.5251	2.0870
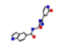	1	25	-5.2926	2.0611
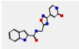	1	26	-5.3958	2.7439
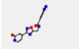	1	27	-5.6126	1.2346
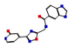	1	28	-5.3118	1.4141
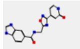	1	29	-5.3391	1.9499
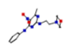	1	30	-5.9148	2.5268
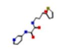	1	31	-5.3972	0.9700
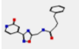	1	32	-5.8289	1.5352
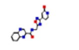	1	33	-5.7849	1.9553
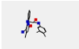	1	34	-5.2345	2.0580
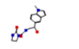	1	35	-5.4719	1.5701
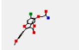	1	36	-5.9897	0.6107

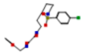	1	37	-5.8395	2.8897
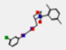	1	38	-5.9174	2.2036
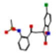	1	39	-5.3516	3.3576
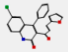	1	40	-5.3087	2.1744
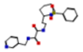	1	41	-5.9877	1.2892
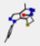	1	42	-4.6787	1.1188
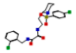	1	43	-5.9380	1.5750
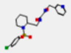	1	44	-5.7951	2.5540
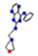	1	45	-5.4850	2.5859
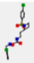	1	46	-5.8528	2.5404
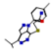	1	47	-4.8824	1.9065
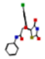	1	48	-4.9395	1.9232
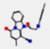	1	49	-5.4735	1.9172
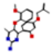	1	50	-5.4986	1.4529
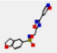	1	51	-5.6931	1.9143
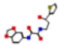	1	52	-5.3251	1.3564
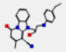	1	53	-5.9215	3.2073

4. ADMET prediction

Table S4. Prediction of ADMET properties of candidate compounds.

NO	Absorption		Distribution		Metabolism		Excretion	Toxicity
	HIA	$F_{20\%}$	BBB	PPB	CYP2D6	CYP3A4	CL	ROA
F1739-0081	0.010	0.011	0.237	0.985	0.445	0.932	9.210	0.015
F2571-0016	0.005	0.033	0.093	0.978	0.120	0.932	3.385	0.019

HIA, Human intestinal absorption; $F_{20\%}$, 20% Bioavailability; BBB, Blood-brain barrier penetration; PPB, Plasma protein binding; CL, Clearance; ROA, Rat oral acute toxicity. CYP2D6 and CYP3A4, The output value represents the predicted probability of being an inhibitor.

Table S5. Drug similarity and synthetic feasibility of candidate compounds.

NO	Lipinski's rule	Pfizer's rule	Golden Triangle
F1739-0081	Accepted	Accepted	Accepted
F2571-0016	Accepted	Accepted	Accepted

5. Activity validation

5.1 Materials

The study utilized Dulbecco's Modified Eagle Medium (DMEM, Shanhai, China, Viva Cell, Catalog No. C3060), Fetal Bovine Serum (ExCell Bio, Shanhai, China, Catalog No. FSP500), and Penicillin/Streptomycin (100×; Logan, US, Hyclone, Catalog No. SV30010). Phosphate Buffered Saline (PBS, US, Utah, Hyclone, Catalog No. SH30256) was also used. A549 cells were sourced from the Shanghai Cell Bank, Chinese Academy of Sciences (Catalog No. SCSP-502). Additionally, the 3-(4,5-dimethylthiazol-2-yl)-2,5-diphenyl tetrazolium bromide (MTT) cell proliferation and cytotoxicity detection kit (Beyotime, Shanhai, China, Catalog No. C0009S) was employed. Ceritinib and Lorlatinib, an FDA-approved ALK inhibitor, was purchased from Aladdin as a positive control compound.

5.2 Instruments

The following instruments were used in the study: a micropipette (Thermo, Waltham, US), a biosafety cabinet (NMAIRE, Guangzhou, China, model NM-425-400S), and a CO_2 incubator (NMAIRE, Guangzhou, China, model NM-5800). For intercellular observation, an inverted fluorescence microscope (Mingmei, Guangzhou, China, model MF53-N) was employed. A refrigerated high-speed centrifuge (Thermo, Waltham, US, model Legend Micro 17R) and a constant temperature water bath (Shanghai Benting Instrument Co., Ltd., Shanghai, China, model BK-3D) were also utilized. To measure absorbance, a high-end microplate reader (Molecular Devices, California, USA, model SPECTRAMAX PARADIGM) was used. Additionally, a confocal microscope (Leica, Wetzlar, Germany, model SP8) was employed for advanced imaging.

5.3 Cell Culturing

The selected cell line was retrieved from cryopreservation and rapidly thawed in a 37 °C water bath with gentle agitation until the cryoprotectant was completely dissolved. After thawing, the cells were transferred to a centrifuge tube containing 5 mL of culture medium and centrifuged at 1000 rpm for 5 minutes. The supernatant was discarded, and the cell pellet was resuspended in complete culture medium supplemented with 10% fetal bovine serum, ensuring thorough mixing for uniform distribution. The cell suspension was then inoculated into a culture dish and incubated at 37 °C in a humidified atmosphere containing 5% CO_2. Once the cells reached the logarithmic growth phase and showed healthy growth

characteristics, they were seeded into a 96-well culture plate at a density of 4×10^3 cells per well and incubated overnight under the same conditions. To maintain humidity, 100 μL of sterile PBS was added to the wells adjacent to each cell culture well.

5.4 Compounds Pre-treatment and Administration Procedures

Candidate Compounds were filtered and sterilized sequentially, then stored at 4 °C for later use. After 24 hours of cell adhesion and growth in the 96-well plates, the culture medium was removed, and the cells were washed twice with PBS buffer. Solutions of each compound, prepared at the desired working concentrations, were then added to the wells. Following compound addition, the cells were incubated at 37 °C in a humidified atmosphere containing 5% CO_2 for 48 hours before proceeding with the subsequent assays.

5.5 MTT Colorimetric Assay

The MTT reagent kit was thawed at room temperature, and 10 μL of the MTT solution was carefully added to each well. The plate was then incubated in a cell culture incubator for 4 hours. Following incubation, 100 μL of Formazan solution was added to each well, gently mixed, and incubated further in the cell culture incubator until complete dissolution of Formazan was observed under an optical microscope. Absorbance values were then measured at 570 nm using a microplate reader to assess cell viability.

The activity results are as follows:

Table S6. Blank and control

name	control	blank
A	2.1019	0.1588
B	2.0761	0.1632
C	2.0455	0.1612
Average	2.0745	0.161067
Correct	1.9134	
Cell Viability (%)	100	

Table S7. Compound F1739-0081 (A549)

name	500μM	300μM	100μM	50μM	10μM
A	0.5275	1.0719	1.6776	1.9929	2.0672
B	0.5327	1.1881	1.5432	2.0997	2.1412
C	0.6558	1.1081	1.6671	2.016	2.0915
Average	0.572	1.1227	1.6293	2.0362	2.099967
Correct	0.410933	0.961633	1.468233	1.875133	1.9389
Cell Viability (%)	21.47623	50.25696	76.73292	97.99836	101.3309

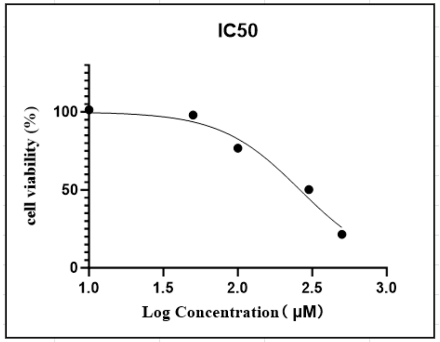

IC_{50}=261.7 μM

Table S8. Compound F2571-0016 (A549)

name	500μM	300μM	100μM	50μM	10μM
A	1.9323	1.9858	2.0397	2.1202	2.1733
B	1.8592	1.9182	2.0609	2.0499	2.1023
C	1.9336	2.0492	2.086	2.0289	2.1693
Average	1.908367	1.9844	2.0622	2.066333	2.1483
Correct	1.7473	1.823333	1.901133	1.905267	1.987233
Cell Viability (%)	91.31753	95.29119	99.35718	99.57319	103.8569

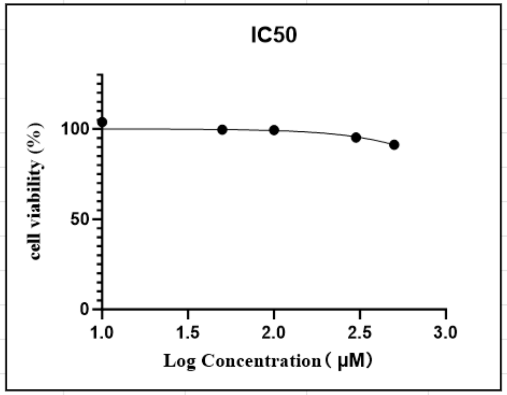

IC_{50}>500μM

Table S9. Ceritinib (A549)

name	500μM	300μM	100μM	50μM	10μM
A	0.1792	0.1765	0.164	0.1663	2.107
B	0.1923	0.1826	0.2033	0.1877	2.1504
C	0.2162	0.1944	0.2031	0.1897	2.1494
Average	0.1959	0.1845	0.190133	0.181233	2.1356
Correct	0.034833	0.023433	0.029067	0.020167	1.974533
Cell Viability (%)	1.820462	1.224675	1.519084	1.053952	103.1932

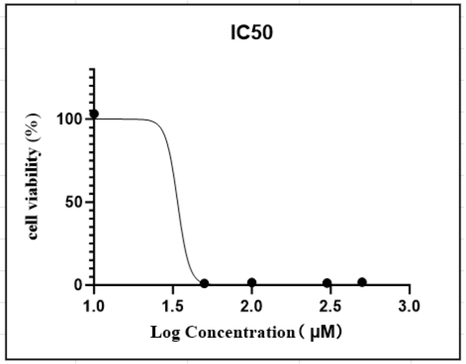

IC50=33.85 μM

Table S10. Lorlatinib (A549)

name	500μM	300μM	100μM	50μM	10μM
A	0.1944	0.8861	1.9187	2.0641	2.0941
B	0.2587	0.8253	2.0447	2.1558	2.2102
C	0.204	0.85	2.0047	2.0806	2.112
Average	0.219033	0.8538	1.989367	2.100167	2.138767
Correct	0.057967	0.692733	1.8283	1.9391	1.9777
Cell Viability (%)	3.029458	36.20368	95.55076	101.3414	103.3587

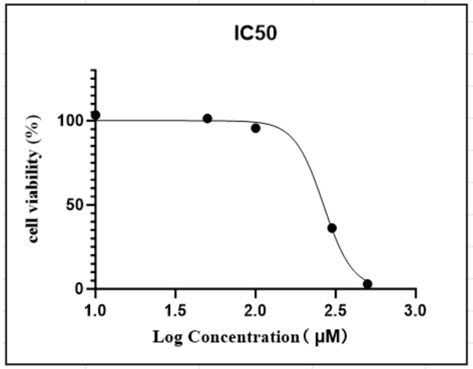
IC50
cell viability (%)
100
50
0
1.0
1.5
2.0
2.5
3.0
Log Concentration (μM)

IC_{50}=265.6 μM
